# All-dielectric polarization-independent optical angular filter

**DOI:** 10.1038/s41598-017-16837-w

**Published:** 2017-11-29

**Authors:** Qinyu Qian, Changqing Xu, Chinhua Wang

**Affiliations:** 1 College of Physics, Optoelectronics and Energy & Collaborative Innovation Center of Suzhou Nano Science and Technology, Soochow University, Suzhou, 215006 China; 2 Key Lab of Advanced Optical Manufacturing Technologies of Jiangsu Province and Key Lab of Modern Optical Technologies of Education Ministry of China, Soochow University, Suzhou, 215006 China

## Abstract

We report on an all-dielectric, polarization-independent angular filter with one-dimension (1D) photonic crystal (PC) composed of semiconductor compatible Si/SiO_2_ pairs. The near-symmetric directional band gap of *p*- and *s*-polarized components and Fabry-Pérot (F-P) resonances are utilized to realize efficient polarization-independent angular filtering for normal incidence. The proposed angular filter is designed and experimentally demonstrated in a large area (5 cm × 5 cm) with multilayer sputtering depositions. Experimental measurements show that a divergence angle of the polarization-independently transmitted beam through the angular filtering sample at 1550 nm is 2.2° only and the transmission is as high as 0.8 at normal incidence. The proposed and demonstrated angular filter suggests an effective way to design and implement semiconductor compatible, all-dielectric and polarization-independent angular filters in a fashion of simple structure and easy-fabrication, which is expected to have great applications in lighting, beam manipulation, optical coupling and optical communications.

## Introduction

Full control of light has always been an important subject in optics. An electromagnetic wave can be characterized by its phase, amplitude, frequency, polarization, and propagating direction. Tremendous work has been done to manipulate amplitude^1,2^, phase^1–4^, frequency^5,6^ and polarization^7,8^. Direction filter (or angular filter) has also been widely studied and is still an important topic today. Zero-index-materials (ZIMs) have been explored for the angular filtering. Ideal ZIMs can filter all non-perpendicular incident waves due to vector matching between ZIMs and surrounding materials. Epsilon-near-zero materials (ENZs) have a permittivity near zero, resulting in a near-zero refractive index, thus are usually utilized to realize ZIMs. Nanowire and multilayer metamaterials made of plasmonic metals are the two most common designs for materials with ENZ^9–12^. Alekseyev *et al*. proposed an array of silver nanowires grown in an anodic alumina membrane to realize an angular filter for *p*-polarized (electric field oriented in the incident plane) incidence at wavelength of 600 nm with a filtering angle of 20° and a very low transmission (0.12 only) due to inherently impedance mismatch and plasmonic metal loss^12^. It is also noted that ENZs can work only for one of either *p*- or *s*-polarized incidence (electric field oriented perpendicular to the incident plane)^9–12^, which greatly limits its applications.

Photonic crystals (PCs) with Dirac-like conical dispersions (DLCDs) can also be used as an angular filter for specific polarizations^13–17^, in which PCs with DLCDs can be approximated as a double zero medium (DZM), i.e., permittivity and permeability approach zero simultaneously at the frequency of Dirac-like point. In 2013, Moitra *et al*. reported a DZM that consists of rods of 10 alternating Si/SiO_2_ layers. The DZM shows good angular filtering with high transmission within an angle of 30°^14^. However, the DZM also works only for TM polarization, and the fabrication of multilayer Si/SiO_2_ rods with a high aspect ratio of 3 μm/0.26 μm (height/width) is difficult, especially in large area. In 2014, Shen *et al*. proposed a 1D PC with varied periodicity to transmit plane waves of specific incident angle (Brewster’s angle) and reflect incident waves of other incident angles^18,19^. However, a specific liquid of certain permeability and permittivity must be used to immerse the device in order to improve its low efficiency. It is also noted that the device can work only at incline incidence at Brewster’s angle (as opposed to conventional normal incidence) and, same as other angular filters, works only for *p*-polarized incidence.

Polarization-independent angular filtering remains still unrealized because of the difficulty in physics in which *s*- and *p*-polarized incident waves exhibit significant difference under off-normal incidences. In this work, we proposed and experimentally demonstrated a semiconductor compatible, all-dielectric and polarization-independent 1D PCs to achieve angular filtering for normal incidence. Unlike numerous studies of angular filtering with either ENZs or DZMs (both are of near-zero refractive index and polarization sensitive), the proposed polarization-independent angular filter (PIAF) shows no character of near-zero refractive index but employs a near-symmetric band structure of *p*- and *s*-polarized components near the band edge to realize angular filtering of normal incidence. By engineering the frequency of the band edge, omnidirectional gaps above the light line except the *k*_y_ = 0 point (i.e., the normal incident angle, the only angle at which the incident light can propagate) can be obtained, and high transmission of the filtered light is ensured by the optimization of F-P resonance in the 1D structure. Experimental results demonstrate the polarization-independent and efficient filtering behavior, in which the transmission is as high as 0.80 and the divergence angle of the transmitted beam is only 2.2° at the designed wavelength of 1550 nm.

## Results

**Figure 1:**
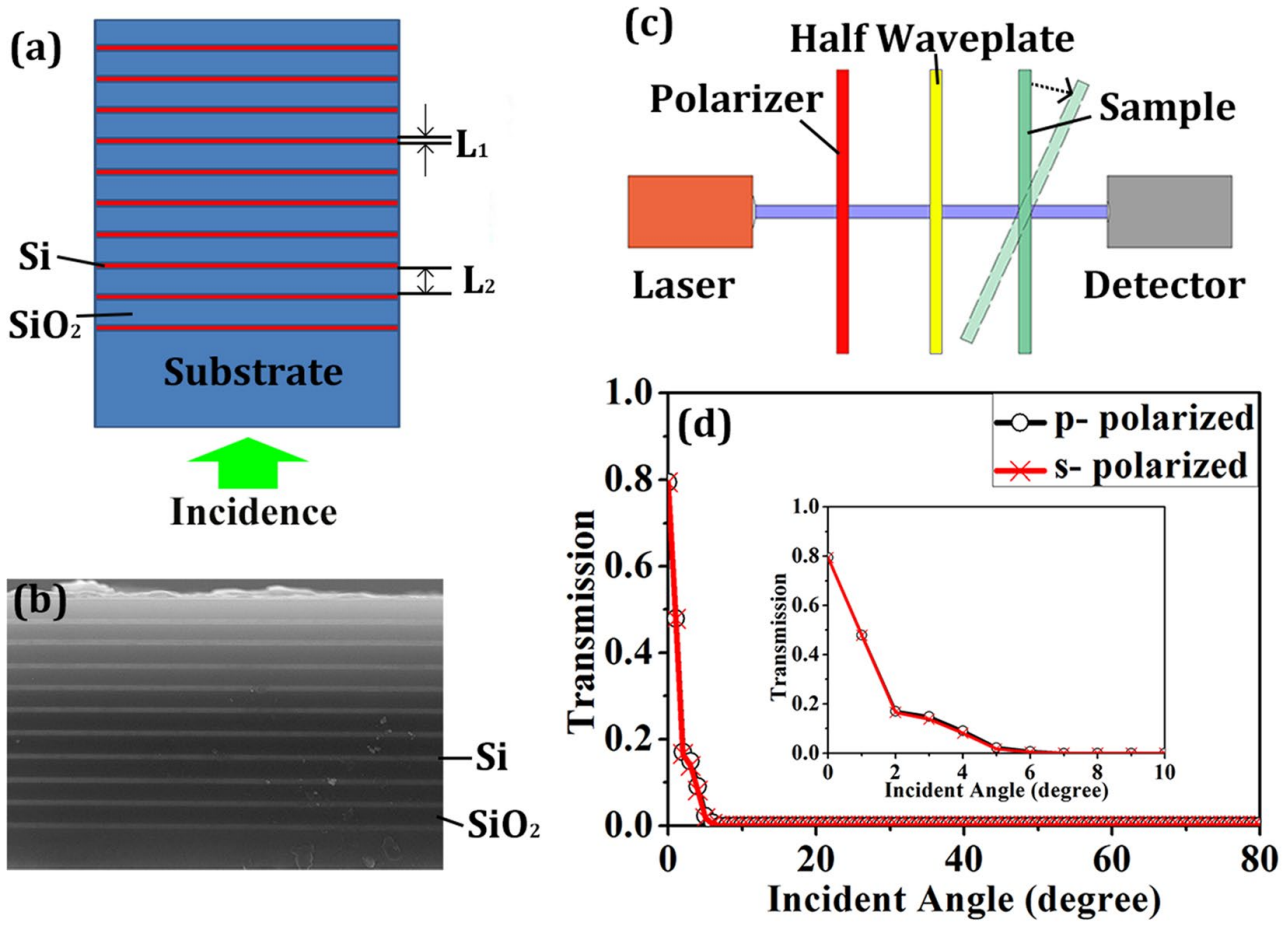
Structure of proposed PIAF and experimental measurements. (**a**) Schematic diagram of the proposed 1D all-dielectric PIAF. (**b**) SEM photograph of the fabricated PIAF. Si layers: light color; SiO_2_ layers: dark color. Thicknesses of these layers are *L*_1_ = 80 ± 8 nm and *L*_2_ = 454 ± 32 nm, respectively. (**c**) Diagram of measurement setup. (**d**) Measured transmission at incident angles from 0° to 80° at 1550 nm. Inset is the amplified experimental transmission at incident angles from 0° to 10°.

Figure 1(a) shows a 1D PIAF working at the wavelength of 1550 nm. The unit cell is constructed by alternative silicon (Si) and silicon dioxide (SiO_2_) layers with thicknesses of *L*_1_ = 80 nm and *L*_2_ = 454 nm, respectively. The electromagnetic wave is incident from the substrate (SiO_2_) as shown in Fig. 1(a). Si and SiO_2_ coatings were alternately deposited on SiO_2_ substrate of 5 cm × 5 cm by ion sputtering. Figure 1(b) shows the SEM photograph of the fabricated 1D all-dielectric PIAF. A near-infrared laser (Agilent Technologies, 81960 A, wavelength tunable from 1503 nm to 1632 nm) and a detector (Thorlabs, PAX5710IR1-T) are used to detect the transmission (Fig. 1(c)). A polarizer and a half waveplate are used to tune the orientation of polarization. The transmission of the incident beam at the designed wavelength of 1550 nm is measured when the PIAF is tilted at different angles as shown in Fig. 1(c). Figure 1(d) shows the angular filtering performance of our fabricated PIAF in that the transmission is 0.8 at normal incidence but falls rapidly to 0.17 and zero when incident angle increases to 2° and 5°, respectively. It is also seen that our fabricated PIAF shows the same angular filtering performance for *p*- and *s*-polarized incidences, which clearly indicates the polarization independent behavior.

**Figure 2:**
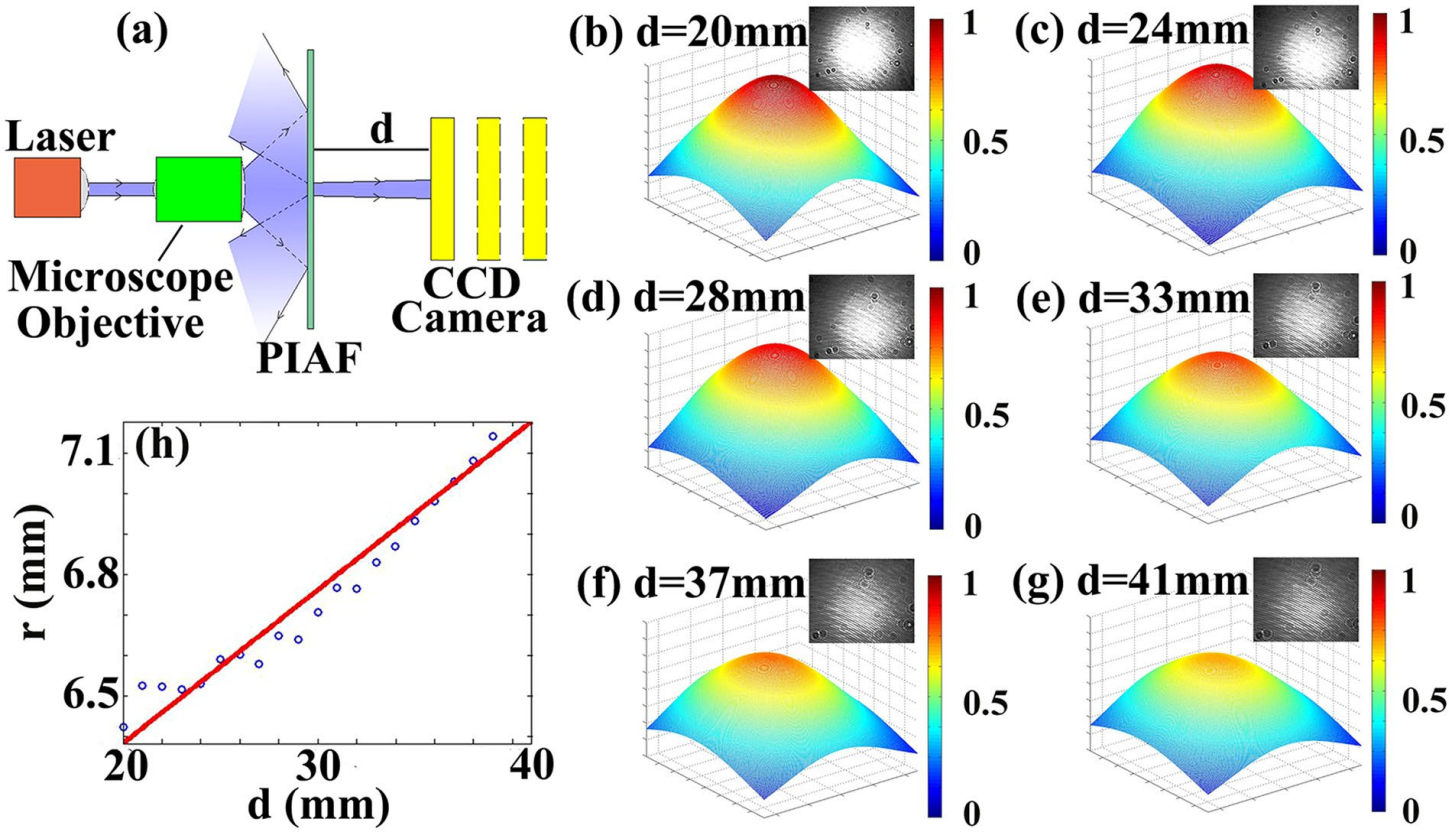
Angular divergence measurement of the fabricated PIAF. (**a**) Diagram of the divergence measurement. (**b–g**) Six photographs taken at different distances away from the PIAF sample from 20 to 41 mm and the corresponding fitted Gaussian profiles. (**h**) Linear fitting of r at different locations.

To further demonstrate the angular filtering effect, divergence behavior of a diffused laser beam transmitting through the PIAF was measured using the experimental setup shown in Fig. 2(a). The laser beam is firstly expanded by a microscope objective (20×) and then passes through the PIAF. The transmitted beam spot at different locations from the PIAF is recorded by a infrared camera (XENICS, XEVA-1.7–320, 320 × 256 pixels). Figure 2(b) shows the photographs of the transmitted beam spot and the corresponding quantitative fitting plots using software MATLAB. Excellent angular filtering can be obtained in terms of the observed beam spots. Quantitative evaluation of beam size of the transmitted light at different locations can be obtained by using three-dimensional fitting of these spots to Gaussian beam profile as follow:

1 $$I=A\,\exp [-\frac{{(x-B)}^{2}+{(y-D)}^{2}}{{C}^{2}}].$$

where *I* is the intensity (grayscale value in the photographs), *x* and *y* represent the coordinate of the beam spot in the photography. *A*, *B*, *C* and *D* are the fitting parameters, in which *A* is the largest grayscale value in the paragraph, *B* and *D* are the *x*-coordinate of the columns and the *y*-coordinate of the rows where the sum of the grayscale value of each pixel is largest, respectively. Figure 2(b–g) are the fitted Gaussian profiles of the experimental beam spots from 20 mm to 41 mm away from the PIAF with a step of 1 mm (only six of them are shown). The fitted beam size *r* (defined as the distance between the center of spot where the intensity is the largest and the position where the intensity falls to 1/*e* ^2^ of that in the center, $r=\sqrt{2}C$) at different locations are given in Fig. 2(h). The slope of the line in Fig. 2(h) represents the divergence angle of the transmitted beam. Linear fitting from Fig. 2(h) can be performed and the following relations can be obtained: *r* = 0.0379*d* + 5.6240 where *d* is the distance from the PIAF to the CCD camera (shown in Fig. 2(a)). The divergence angle of the transmitted beam can be calculated as *θ* = *arctan* 0.0379 ≈ 2.2°, which is well consistent with the previous results (Fig. 1(d)).

## Theory

**Figure 3:**
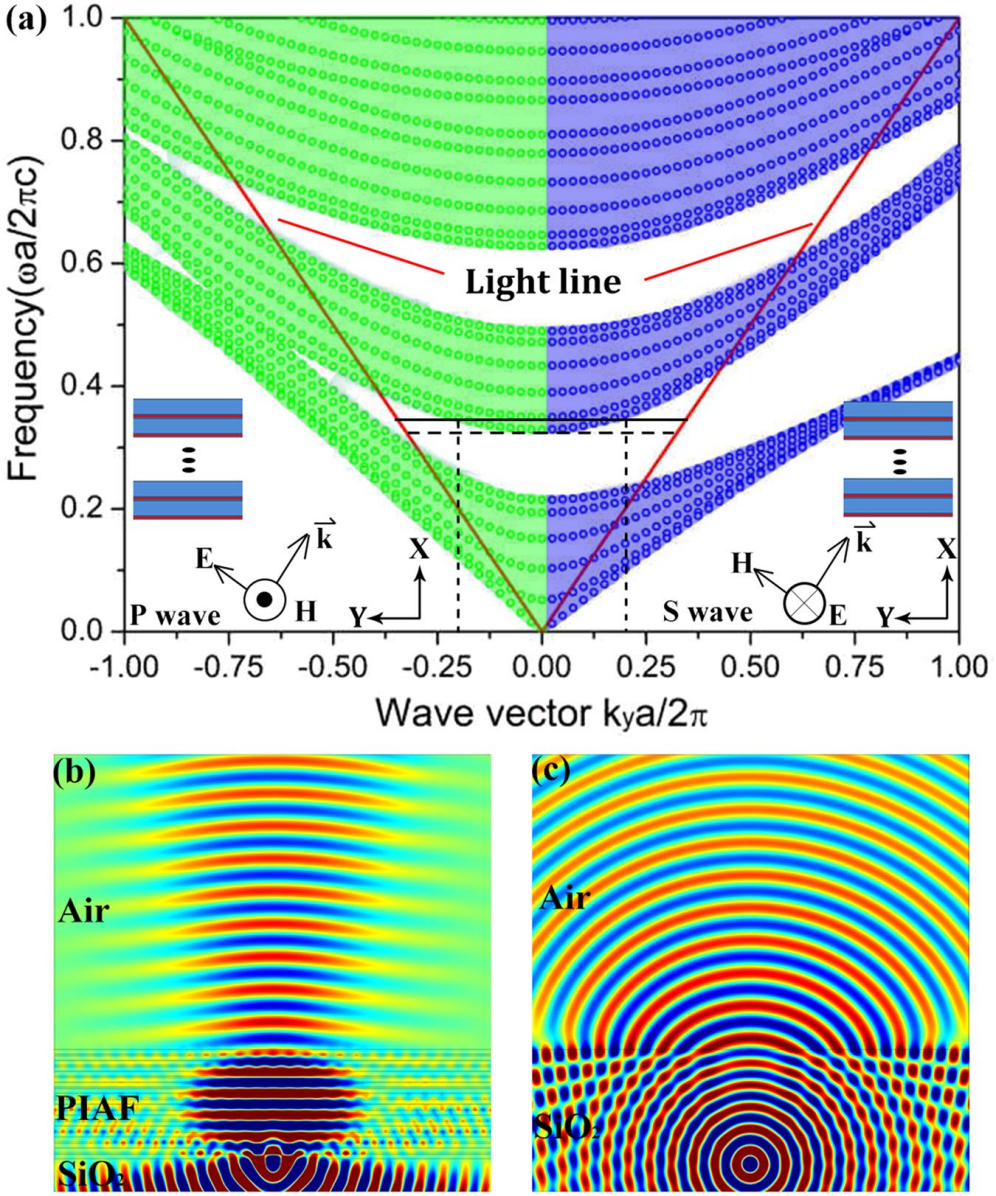
Theoretical design and analysis of the proposed PIAF. (**a**) The projected band structure of *p*- and *s*-polarized waves. The red lines represent the light line. The black horizontal line represents the wavelength of 1550 nm, the dashed horizontal line represents the band edge frequency (*ω* = 0.33 (2*πc*/*a*), i. e., wavelength of 1618 nm) at *k*_y_ = 0. (**b**) Field distribution when a point source is placed under the PIAF; and (**c**) Field distribution when a point source is placed under a SiO_2_ bulk as a comparison. The external medium is set as air and the wavelength is 1550 nm.

Theoretical design and analysis can be performed based on the unit cell of the proposed PIAF formed by dielectric layers of high and low refractive index whose thicknesses are, respectively, *L*_1_ = *ma* and *L*_2_ = *na*, where *a* is the lattice constant and *m* + *n* = 1. The generalized design process is as follows: Firstly, two dielectric materials of high and low refractive index and optically lossless at the working wavelength are selected. At designated working wavelength of 1550 nm in our case, Si and SiO_2_ (the refractive index of Si and SiO_2_ is 3.48 and 1.44, respectively) are chosen. Then *L*_1_ and *a* with *L*_2_ = *a* − *L*_1_ are adjusted to tune the position of the band edge from which the working wavelength can be mainly determined. It is noted that when either *L*_1_ (accordingly *L*_2_ = *a* − *L*_1_ with fixed *a*) or the layers of the unit cell can affect the wavelength position of the transmission peak. In our proposed PIAF, the optimized parameters are *L*_1_ = 0.15*a* and *L*_2_ = 0.85*a*, respectively, with *a* = 534 nm. The incident electromagnetic wave with a wave vector $\overrightarrow{k}={k}_{x}\hat{x}+{k}_{y}\hat{y}$ can be either *p*- or *s*-polarized waves. For the *s*-polarized wave, the electric field is perpendicular to the *x*-*y* plane, as is the magnetic field for the *p*-polarized wave (inset in Fig. 3(a)). The projected band structure of *p*- and *s*-polarized waves are shown in Fig. 3(a). The green and blue areas indicate the propagating states of *p*- and *s*-polarized wave incidence, respectively. The white areas represent regions of the directional band gap. Those states in the band gap can propagate in the homogeneous medium but will decay in the PIAF. The red line represents the light line. Above the light line, incident waves from substrate can freely propagate in the PIAF. Below the light line, there exist evanescent modes that cannot reach the PIAF from a faraway source. It is noted that in the first band gap there exist an omnidirectional reflection region, which is defined between the normal incidence band edge and light line^6,20^. Above but near (i. e., black line in Fig. 3(a), corresponding to wavelength 1550 nm) the band edge frequency (i. e., *ω* = 0.33 (2*πc*/*a*), wavelength 1618 nm, horizontal dashed line in Fig. 3(a)), the eigenstate in the PIAF distributes near *k*_y_ = 0, which indicates that the near-normally incident waves can pass through the PIAF sample. For *k*_y_ ≠ 0, the transmittance will decrease quickly due to the existence of directional band gap. Therefore, such a 1D PC structure can be applied to achieve angular filtering, i. e., only the normal incident waves can transmit through the PC structure, while all other incident waves will be reflected due to the existence of directional band gap. It is also noted that the band diagram of *p*- and *s*-polarized wave above the light line in Fig. 3(a) shows excellent symmetric behavior, which provides solid physical evidence for the polarization independent angular filtering of the proposed PIAF. The angular filtering effect through the PIAF can also be visually observed from the numerical simulation shown in Fig. 3(b). In Fig. 3(b), a point source is placed right under the PIAF. It is seen that the PIAF structure filters effectively the wave components with large *k*_y_, while the near-normally incident electromagnetic waves can pass through the PIAF with a small divergence angle. In contrast, when the PIAF is replaced by a pure bulk SiO_2_ (Fig. 3(c)), it is seen that the transmitted waves of the point source propagate along all directions (spherical waves), as expected, with a wave transform across the interface from SiO_2_ to air for all of wave components. It should be noted that in Fig. 3(b) and (c), the fields under the PIAF and the reference SiO_2_ are the total fields resulting from the superposition of incident and reflected light, the fields above the PIAF and the reference SiO_2_ are the pure transmitted light. It is thus seen that the fields in the “incident space” (lower part of the Figure) is much stronger than those in the “transmission space” (upper part of the Figure). The transmission under “normal incidence” through the PIAF is, in fact, very high, ~0.92 at wavelength 1550 nm in simulation.

**Figure 4:**
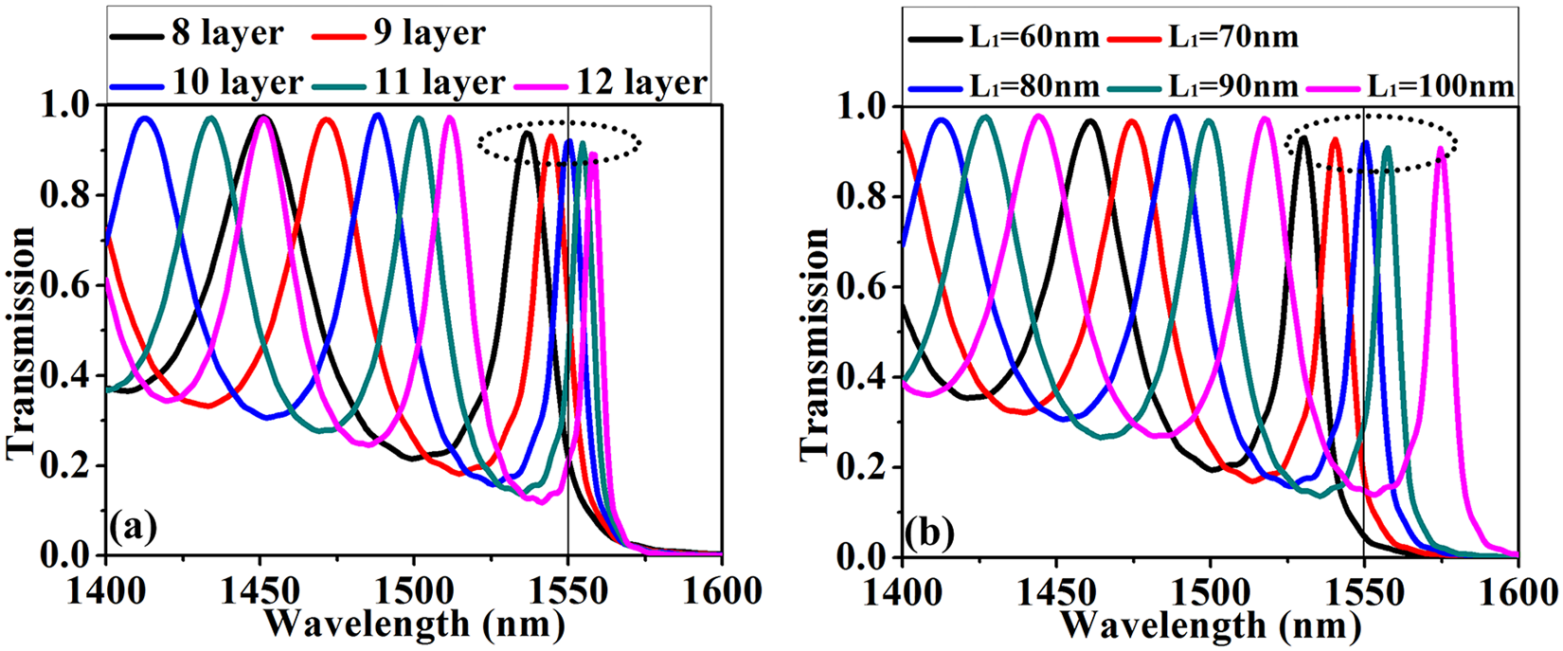
Transmission spectrum of the proposed PIAF. (**a**) Transmission spectrum with different layers of unit cell at normal incidence. (**b**) Transmission spectrum with different thicknesses of Si and SiO_2_ in each unit cell. The number of unit cell is fixed to 10 and total thickness of unit cell is fixed to *L*_1_ + *L*_2_ = 534 nm.

The transmission under normal incidence through the PIAF, however, is usually very low due to the mismatched impedance of the 1D PC structure and background media. To increase the energy efficiency, F-P effect can be utilized for realizing high transmission and sharp resonant peaks in the transmission spectra. The F-P resonant peaks can be engineered by utilizing the scaling properties of Maxwell’s equations^20^. If the linear dimensions of the structures in a given PC are scaled uniformly by a factor of *α*, the frequency *ω* and wave vector *k* should also be scaled according to the relations $\omega ^{\prime} =\omega /\alpha$ and $k^{\prime} =k/\alpha$ ^21^. The transmission spectrum for the PIAF structure with different layers of unit cell (8~12 layers) are plotted in Fig. 4(a). The transmission is simulated using the finite difference time domain method (Lumerical FDTD Solutions, Canada). In the simulation, *L*_1_ = 80 nm and *L*_2_ = 454 nm are used. It is seen from Fig. 4(a) that the F-P peaks redshifts and the bandwidth decreases as the layers of Si/SiO_2_ pairs increase. Only the rightmost peak can be employed in designing the angular filter because it is the nearest to the band edge. This behavior can be used to tune the working wavelength, as seen in Fig. 4(a) that working wavelength at 1550 nm (black vertical line) can be obtained with a 10-layer PIAF structure with *L*_1_ = 80 nm and *L*_2_ = 454 nm. The rightmost peak can be tuned in the range from 1535 nm to 1558 nm (indicated with the peaks in dotted circle in Fig. 4(a)).

The F-P resonant peaks can also be tuned in a wider range by changing the thickness of Si and SiO_2_ (*L*_1_ and *L*_2_) in unit cell (Fig. 4(b)) because the band edge is changed when changing *L*_1_ and *L*_2_ (in contrast, the band edge is unchanged when changing the number of layers), in which the changing of *L*_1_ and *L*_2_ (with fixed *a* = *L*_1_ + *L*_2_ = 534 nm) will result in the change of permittivity distribution. When the permittivity distribution in the unit has a small perturbation from $\varepsilon (r)$ to $\tilde{\varepsilon }(r)$, the eigenfrequency will be shifted from ${\omega }_{nk}$ to ${\tilde{\omega }}_{nk}$ according to the formula^22,23^:

2 $${(\frac{{\tilde{\omega }}_{nk}}{{\omega }_{nk}})}^{2}-1\approx \frac{\int [\varepsilon (r)-\tilde{\varepsilon }(r)]{|{E}_{nk}(r)|}^{2}dr}{\int \varepsilon (r){|{E}_{nk}(r)|}^{2}dr}$$

where ${E}_{nk}(r)$ denotes the field distribution of unperturbed modes. By the guidance of Eq. (2), we can adjust the thickness of silicon (while the total thickness of silicon and silica is fixed) to tune the eigenfrequency (working wavelength). Figure 4(b) demonstrates the tuning capability of transmission peaks for normal incident waves near 1550 nm with a 10-layer PIAF structure. When the thickness of silicon (*L*_1_) layer changes from 60 nm to 100 nm, the wavelength of the peak nearest to the band edge can be tuned from 1530 nm to 1575 nm (indicated in the dotted circle in Fig. 4(b)), and the peak at wavelength of 1550 nm can be obtained with *L*_1_ = 80 nm (vertical line in Fig. 4(b)). It is seen from Fig. 4(b) that the F-P peaks redshifts and the bandwidth decreases as *L*_1_ increases (*L*_2_ decreases). It should be noted that different combinations of thickness of Si and SiO_2_ and also the number of unit cell can be used to obtain a similar performance of the PIAF. The transmission peaks and the corresponding wavelength positions in Fig. 4 can also be calculated and understood based on multi-layer thin film system by transfer matrix method. The detailed calculation and discussion is given in Supplementary Information.

**Figure 5:**
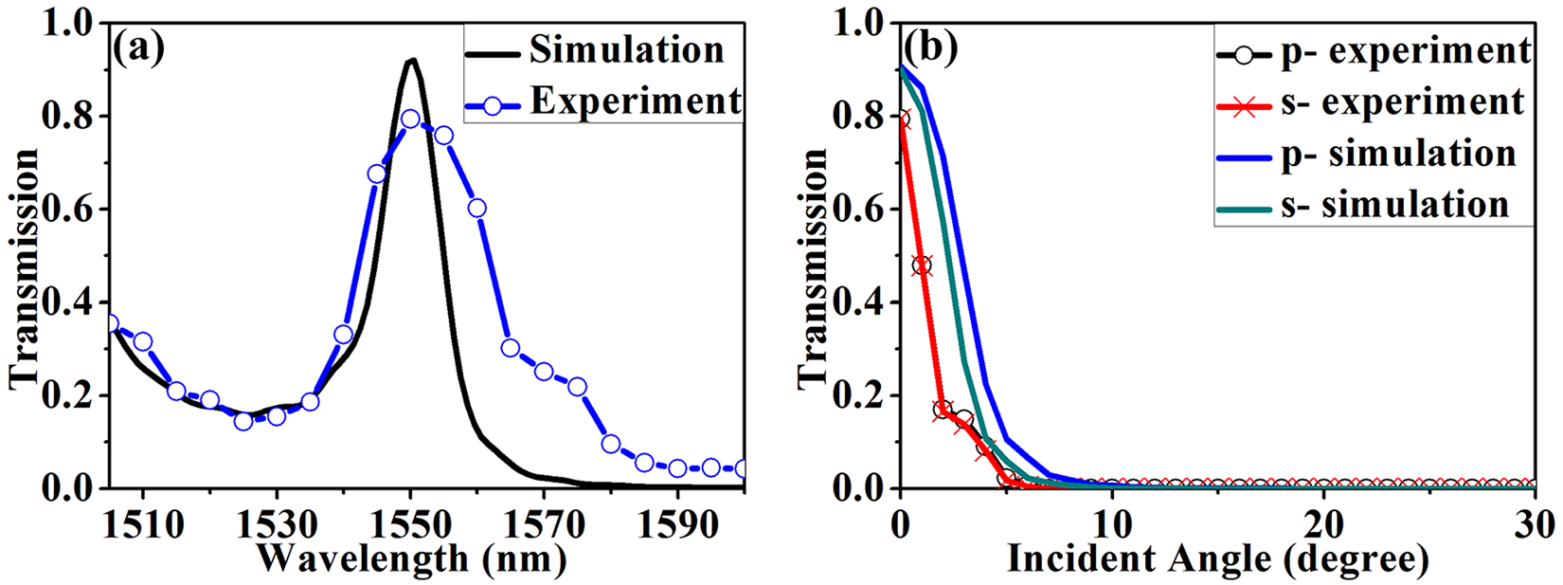
Comparison of simulated and experimental transmission spectrum of PIAF. (**a**) Transmission spectrum at normal incidence. (**b**) Transmission spectrum different incident angles from 0° to 30° at 1550 nm. The number of layers is 10.

The detailed comparison of the performance between the fabricated PIAF (*L*_1_ = 80 ± 8 nm and *L*_2_ = 454 ± 32 nm) and the theoretical (*L*_1_ = 80 nm and *L*_2_ = 454 nm) results are given in Fig. 5. Figure 5(a) and (b) show the comparison of experimental and simulated transmission at normal incidence and at different incident angles from 0° to 30° at wavelength 1550 nm, respectively. It is seen that the experimental results agree well with theoretical simulation. The small deviations in transmission peak and bandwidth in Fig. 5(a) and divergent angle in Fig. 5(b) between the experiment and theory can be mainly attributed to fabrication errors of the device, in which the thickness of each Si/SiO_2_ layer may deviate from the designed values (the deviation is typically ~5% from the designed value). To verify the effect of the thickness of each layer on the performance of the transmission (i.e., peak transmission, peak wavelength and bandwidth in Fig. 5(a)), detailed simulations have been performed when the thickness of each layer randomly deviates from the designed value. The simulation results confirm that all the peak transmission, peak wavelength and the bandwidth change when the thickness of each layer deviates from the designed value. The deviation of the thickness of each layer also changes the position of the band edge. It can be seen from Fig. 3(a) that the divergent angle decreases when the band edge moves up, leading to a smaller divergent angle in the experiment than that in the simulation, as witnessed in Fig. 5(b). The transmission spectrum under *p*- and *s*- polarized incidences almost coincides, which excellently demonstrates polarization-independent angular filtering of the fabricated PIAF.

## Discussion

In conclusion, we proposed and experimentally demonstrated an all dielectric angular filter working for incident waves of arbitrary polarization for the first time. An omnidirectional reflection region between the band edge and light line is utilized to reflect off-normally incident waves and transmit near-normally incident waves, and optimized F-P resonance is employed to realize high transmission. The filtering angle at a desired wavelength can be tunable with specified design in accordance with practical requirement. The proposed semiconductor-compatible all dielectric PIAF is easy to fabricate in large area and the working wavelength can be tuned easily by optimizing the number of layers or the fraction of Si and SiO_2_. The working principle of the proposed PIAF can be easily applied in other wavelength bands by replacing the materials with optimized parameters, which provides a generalized and easy way to implement polarization independent angular filters. The demonstrated PIAF is expected to have great applications in lighting, optical coupling and optical communications where directional beam is required with compact and integrated angular filters, and also in beam shaping and filtering in which noise of high spatial frequency, such as scattered light spots or interfering fringes/patterns resulted from diffraction effects, nonlinear effects and defects in optical material and devices, can be effectively eliminated with the proposed angular filter.

## Electronic supplementary material

Supplementary information
