# Supplementary material for: All-dielectric polarization-independent optical angular filter
Source: Sci Rep. 2017 Nov 29;7:16574. doi: 10.1038/s41598-017-16837-w (PMC5707375; doi:10.1038/s41598-017-16837-w)
Supplement: Supplementary file 1 — Supplementary information [file 41598_2017_16837_MOESM1_ESM.pdf]

Supplementary information: All-dielectric polarization-independent optical angular filter

Qinyu Qian[1,2,†], Changqing Xu[1,†] & Chinhua Wang[1,2,*]

[1] College of Physics, Optoelectronics and Energy & Collaborative Innovation Center of Suzhou Nano Science and Technology, Soochow University, Suzhou 215006, China

[2] Key Lab of Advanced Optical Manufacturing Technologies of Jiangsu Province and Key Lab of Modern Optical Technologies of Education Ministry of China, Soochow University, Suzhou 215006, China

[†] These authors contributed equally to this work

Email: chinhua.wang@suda.edu.cn

Analysis of the transmission of the proposed PIAF under normal incidence

The position of F-P peaks can be designed and tuned precisely based on the transmission under normal incidence onto the PIAF. The transmission under normal incidence of the PIAF can be derived by using the method of transfer matrix. The transfer matrix from SiO_2 to Si is:

$$M_1 = \begin{bmatrix} \exp(ik_{Si}\Delta_{Si}) & 0 \\ 0 & \exp(-ik_{Si}\Delta_{Si}) \end{bmatrix} \begin{bmatrix} (1+\frac{k_{SiO_2}}{k_{Si}})/2 & (1-\frac{k_{SiO_2}}{k_{Si}})/2 \\ (1-\frac{k_{SiO_2}}{k_{Si}})/2 & (1+\frac{k_{SiO_2}}{k_{Si}})/2 \end{bmatrix}$$

$$= \begin{bmatrix} (1+\frac{k_{SiO_2}}{k_{Si}})\exp(ik_{Si}\Delta_{Si})/2 & (1-\frac{k_{SiO_2}}{k_{Si}})\exp(ik_{Si}\Delta_{Si})/2 \\ (1-\frac{k_{SiO_2}}{k_{Si}})\exp(-ik_{Si}\Delta_{Si})/2 & (1+\frac{k_{SiO_2}}{k_{Si}})\exp(-ik_{Si}\Delta_{Si})/2 \end{bmatrix} \quad (1)$$

and the transfer matrix from Si to SiO_2 is:

$$M_2 = \begin{bmatrix} \exp(ik_{SiO_2}\Delta_{SiO_2}) & 0 \\ 0 & \exp(-ik_{SiO_2}\Delta_{SiO_2}) \end{bmatrix} \begin{bmatrix} (1+\frac{k_{Si}}{k_{SiO_2}})/2 & (1-\frac{k_{Si}}{k_{SiO_2}})/2 \\ (1-\frac{k_{Si}}{k_{SiO_2}})/2 & (1+\frac{k_{Si}}{k_{SiO_2}})/2 \end{bmatrix}$$

$$= \begin{bmatrix} (1+\frac{k_{Si}}{k_{SiO_2}})\exp(ik_{SiO_2}\Delta_{SiO_2})/2 & (1-\frac{k_{Si}}{k_{SiO_2}})\exp(ik_{SiO_2}\Delta_{SiO_2})/2 \\ (1-\frac{k_{Si}}{k_{SiO_2}})\exp(-ik_{SiO_2}\Delta_{SiO_2})/2 & (1+\frac{k_{Si}}{k_{SiO_2}})\exp(-ik_{SiO_2}\Delta_{SiO_2})/2 \end{bmatrix} \quad (2)$$

where k_{Si} and k_{SiO2} are the wave vectors in Si and SiO_2, and Δ_{Si} and Δ_{SiO2} are the thicknesses of Si and SiO_2 (i.e., L_1 and L_2 in Fig. 1 in the manuscript), respectively.

The transfer matrix of the PIAF of a 10-Si/SiO_2-pair system can then be derived as:

$$T = \left(M_2 M_1\right)^{10} = \begin{bmatrix} T_{11} & T_{12} \\ T_{21} & T_{22} \end{bmatrix} \quad (3)$$

It is noted that the last interface (i.e., the interface between the top SiO_2 and air) is not included in the above transfer matrix T. The total transmission of the PIAF including the top SiO_2 and air is given as:

$$t = 1 - \left| \frac{T_{11}(k_{SiO_2} - k_{air}) - T_{21}(k_{SiO_2} + k_{air})}{T_{22}(k_{SiO_2} + k_{air}) - T_{12}(k_{SiO_2} - k_{air})} \right|^2 \quad (4)$$

where k_{air} is the wave vector in air.

The transmission t calculated by using the above analytical transfer matrix is given in the following Fig. S1(a). As a comparison, the transmission calculated using numerical method (FDTD) directly from the structure of Fig. 1 in the manuscript is given in Fig. S1(b). The multiple peaks observed in Fig. S1 is corresponding to multiple eigenmodes at the band edge, so that different types of F-P resonances (i.e., F-P peaks at different wavelengths) are excited[1].

It is seen that the results calculated using two different methods are well consistent with each other as seen in Fig. S1. The slight difference between the two results, especially in the short wavelength end, is due to the fact that optical loss is not considered in the transfer matrix so that the calculated transmission of using transfer matrix is higher than that of the FDTD simulation (Si has no optical loss in the NIR range but high optical loss in the visible band).

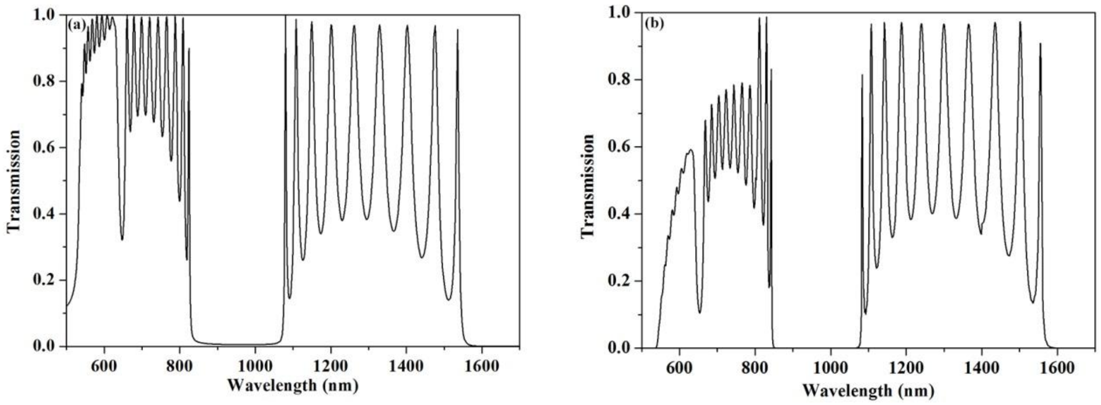

Figure S1 Transmission spectrum of the proposed PIAF (a) calculated by analytical transfer matrix and (b) simulated by numerical FDTD method, respectively.

The calculated results in Fig. S1 are also consistent with Fig. 3(a) in the manuscript, in which a band gap in the frequency range from 0.500 to 0.623 (i.e., wavelength from 857 nm to 1070 nm) under normal incidence in Fig. 3(a), is corresponding to the zero transmission band (i.e., no F-P peaks can be observed in the wavelength band ~850 nm to 1070 nm) in Fig. S1.

The analytical transfer matrix method can be used to analyze and guide the design process of the proposed PIAF, and also the similar filters in which different materials with different dimensions at different wavelengths can be employed and optimized.

1. Xu, C. *et al.* Design of full-*k*-space flat bands in photonic crystals beyond the tight-binding picture. *Sci. Rep.* **5**, 18181 (2015).
